# Alveolar Hemorrhage

**Published:** 1859-08

**Authors:** Geo. Watt


### ALVEOLAR HEMORRHAGE.

BY GEO. WATT.

THOUGH in ordinary cases the extraction of a tooth is followed by but a slight flow of blood, yet few practitioners have failed to witness cases in which the hemorrhage was quite annoying, if not dangerous.

Alveolar hemorrhage may be considered as *accidental* or *constitutional.* Without feeling under obligation to defend the propriety of the use of these terms, we will state that, when the hemorrhage is the result of an undue laceration or incision of the gum and its vessels, we call it accidental—when it results from a morbid state of the blood, or from a peculiar state of the constitution, (called the hemorrhagic diathesis,) we consider it constitutional.

In the treatment of accidental hemorrhage, the appropriate use of styptics and pressure will accomplish all that is desired.

Nature's process for arresting hemorrhage is, to close the bleeding vessels by coagulating the fibrin of the blood—by forming a *clot* in or around the mouth of the vessel. The clot thus formed is in many cases, not firm enough to resist the pressure of the blood, and, hence, the bleeding continues. Art steps in to assist, and, by *pressure*, retains the clot in its place, when, in most cases, it effectually plugs up the vessel. In a good constitution, except where a large vessel is divided, this combination of the powers of art and nature is able to arrest the bleeding. But when the divided vessel is large, the pressure of the blood breaks down (or tears up) the clot, and other means are necessary. And if the blood be abnormal, the clot, if formed at all, may be of so soft a texture that it will avail nothing, even when pressure is appropriately applied to support it.

The object in using styptics is to obtain a firmer clot than is produced by the spontaneous coagulation of the blood. All styptics act chemically. To exert a styptic influence, an agent must have an affinity for some component of the blood; and the compound formed by virtue of this affinity must be, to some extent, insoluble. The albumen and fibrin are the principal constituents of the blood concerned in the combination with styptic agents. Many substances which manifest a strong affinity for these substances have no styptic influence. For example, the caustic alkalies combine energetically with them, but form liquid compounds, and therefore, present no obstruction to the flow of blood.

It is important, then, in deciding the choice of styptic agents, to notice carefully the characteristics of the compounds formed, respectively, by their union with albumen and fibrin. To be better understood, let us suppose a set of experiments with tannin, nitrate of silver and albumen. The nitrate combines energetically with the albumen, forming a firm coagulum or clot; but this coagulum softens and dissolves in albumen. Hence, if more albumen be present than is sufficient to combine with and neutralize the nitrate, the clot can not remain solid and firm.

If, then, the nitrate be applied to a bleeding vessel, a coagulum is formed in its mouth; but the blood in the vessel (containing fresh albumen) gradually disolves its way through it. The arrest of hemorrhage by this agent is, therefore, but temporary. We are thus minute because many infer that, as the nitrate is a powerful escharotic, it is also a most reliable styptic.

On the other hand, if tannin (tannic acid) be applied the coagulum will be less extensive but more firm, while it is not soluble in albumen or any other ingredient of the blood.—Consequently the blood cannot *dissolve* its way through it, as in the former case; and, if it be judiciously supported by pressure, so that the blood cannot *break* through it, the result is an arrest of the bleeding.

Sulphate of copper, sulphate of zinc, acetate of lead, and kindred mineral astringents, also form compounds with albumen and fibrin which are soluble in an excess of albumen, and consequently, in fresh blood. Their styptic power is, therefore, but transient.

In February, 1858, Professor C. had an upper molar extracted, about 10 o'clock in the morning. The hemorrhage was slight at the time; but about 12 M. it became excessive. He applied successively, several of the mineral astringents, with but temporary success. At 8 P. M., I visited him, and found his attendants, by his direction, using the sulphate of copper and pressure, without any diminution of the flow of blood. I removed the application, washed out the mouth, applied a solution of tannin on a pledget of cotton, and confined it by pressure. The bleeding ceased immediately, and did not return. A number of similar cases might be reported, but this must suffice, as our present object is *illustration* not *confirmation.*

The sesqui chloride of iron is a more reliable styptic than tannin. It combines promptly with the albumen and fibrin of the blood as well as with the coats of the vessel; and the compound it forms with them is all that we can expect. In urgent cases we much prefer it to tannin.

The actual cautery (a hot iron) is not a disirable remedial agent in alveolar hemorrhage, nor is it as efficient as other means in our power.

In making pressure for the arrest of alveolar hemorrhage, some judgment is required. Filling the socket will not always answer the purpose. The margins of the gums escape the pressure when it is applied, and may continue to bleed.—It should, therefore, be applied so as to bring them toward each other. This is readily accomplished by cutting a piece of cork in the shape of a block letter V, and setting it astride the gum, after applying to it a fold of cotton, wool, or other soft substance. The closure of the mouth on the cork gives the required pressure; and the mouth should be kept closed by a bandage.

When the hemorrhage arises from constitutional causes, the constitution should be treated; and, when it is known that the patient is of a hemorrhagic diathesis, the treatment should generally precede the operation. If bowels are constipated, a saline cathartic will be advantageous, after which opium and acetate of lead should, generally, constitute the main reliance.

General principles must govern the practitioner in the use of these remedies. Of course, different cases will require different treatment.

If there be no opportunity to treat the constitution before the operation, it should be promptly treated afterward; and at the same time, local treatment should be diligently used. The intelligent operator will seldom have much difficulty, if he has an opportunity to treat the case promptly.

